# Case report: Prenatal diagnosis of rare chromosome mosaicism: discordant results between chorionic villi and amniotic fluid samples

**DOI:** 10.3389/fgene.2023.1165019

**Published:** 2023-06-05

**Authors:** Lingping Li, Xijing Liu, Qinqin Li, Lili Zhang, Yueyue Xiong, Shanling Liu, He Wang, Hongmei Zhu, Xuemei Zhang

**Affiliations:** ^1^Department of Medical Genetics and Prenatal Diagnosis Center, West China Second University Hospital, Sichuan University, Chengdu, China; ^2^Department of Obstetrics and Gynecology, West China Second University Hospital, Sichuan University, Chengdu, China; ^3^Key Laboratory of Birth Defects and Related Diseases of Women and Children (Sichuan University), Ministry of Education, Chengdu, China

**Keywords:** chromosome mosaicism, prenatal diagnosis, nuchal cystic hygroma, monosomy X, genetic testing

## Abstract

**Objective:** We described a unique case of near-negative chromosome mosaicism in chorionic villi but complete monosomy X in amniotic fluid.

**Methods:** Chorionic villus sampling and amniocentesis were performed separately in the first and second trimesters. Chromosomal microarray (CMA) and rapid aneuploidy detection (QF-PCR and FISH) were performed on placental villi and uncultured amniotic fluid. After pregnancy termination, the placenta, umbilical cord, and fetal muscle tissues were sampled for FISH detection.

**Results:** The CMA revealed a lower signal from chromosome X in chorionic villi, with a copy number of 1.85, implying the presence of mosaic monosomy X. However, the QF-PCR and FISH results were nearly normal. In uncultured amniotic fluid, CMA and rapid aneuploidy detection indicated complete monosomy X. Across different sampling points on the aborted fetus, the FISH results varied from normal, to mosaic, and then complete monosomy X.

**Conclusion:** This case presents a rare and complex situation where sampling from uncultured chorionic villi indicated low-level chromosome mosaicism, while sampling from amniotic fluid revealed complete monosomy X. Although some of these discordant outcomes may be due to methodological limitations, we conclude that prenatal consultation should be combined with fetal ultrasound phenotype and genetic testing for a comprehensive evaluation of fetal genetic abnormalities.

## Introduction

Chromosome mosaicism is defined as the presence of two or more genetically distinct cell types in one individual developed from a single zygote. This phenomenon is considered an abnormal chromosomal event stemming from postzygotic errors (Phillips et al., 1996; Grati, 2014; Taylor et al., 2014). The mechanisms of mosaicism include chromosome non-disjunction, anaphase lag, or endoreplication. Fetal chromosome mosaicism is broadly categorized into two types: general mosaicism (presence of two or more cell lines throughout the entire organism) and confined mosaicism. The latter includes confined placental mosaicism (CPM, in which a chromosomally abnormal cell line is restricted to the placenta while the fetal chromosomes are normal) and confined fetal mosaicism (the presence of an abnormal chromosome cell line in a particular area of the fetus) (Taylor et al., 2014; Toutain et al., 2018). Where and when an error occurs from zygote to fetus may cause genetic testing to yield discordant results, depending on if the sample was obtained from chorionic villi (CV), amniotic fluid (AF), or fetal blood (FB). These three sample origins correspond to the predominant testing procedures during prenatal diagnosis: chorionic villus sampling (CVS), amniocentesis (AC), and fetal blood sampling (FBS). Clinically, the most common form of mosaicism is CPM (Phillips et al., 1996; Taylor et al., 2014), whereas the opposite (abnormal fetal chromosome but normal or near-normal placental chromosome) is rare.

Herein, we present a case study of the rarer form, with samples from uncultured chorionic villi indicating a near-normal X chromosome, but the amniotic fluid showing complete monosomy X. We postulated that this case represented low-level mosaicism of X monosomy in the placenta with the fetus exhibiting complete X monosomy and explored the potential causes of these discordant outcomes.

## Case presentation

**FIGURE 1:**
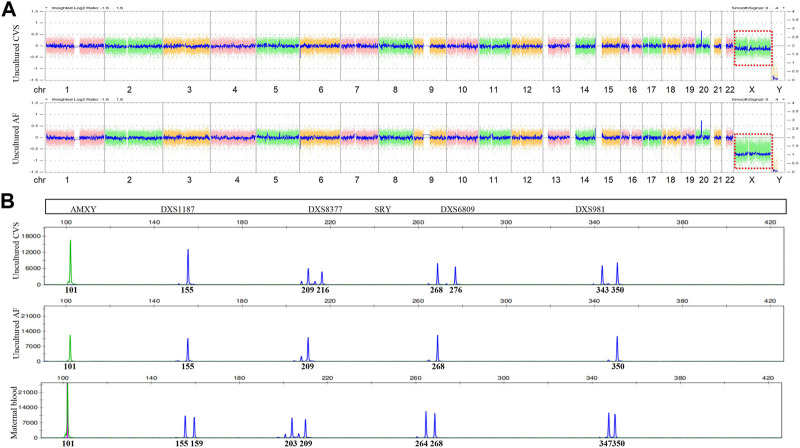
Chromosomal microarray (CMA) and quantitative fluorescent (QF)-PCR results. **(A)** Chromosome X of CMA (red box) in uncultured CVS (upper) and AF (lower). **(B)** Markers on chromosome X of QF-PCR in uncultured CVS (upper), AF (middle), and maternal blood (lower).

A 28-year-old woman (gravidity 1, parturition 0) underwent CVS at 13 + 2 gestational weeks (GW) because the first-trimester ultrasound showed an increased nuchal translucency of 5.5 mm, accompanied by an a-wave reversal in the venous catheter. Genomic DNA was extracted from uncultured CV, then subjected to aneuploidy detection via quantitative fluorescent PCR (QF-PCR) and Chromosomal microarray analysis (CMA) (Affymetrix 750K). The latter method involves array comparative genomic hybridization and detection of single-nucleotide polymorphisms (SNPs). Additionally, genomic DNA extracted from the peripheral blood of this woman was used to test for maternal cell contamination (MCC) (Figure 1).

The results ruled out MCC, while QF-PCR showed that chromosomes 13, 18, 21, X, and Y were nearly normal. The only aberration was a slight deviation in the ratio of short tandem repeats (STR) on chromosome X from reference values (0.86–1.21) (Table 1). In contrast, CMA detected a decreased chromosome X signal with a copy number of 1.85, while the Y copy number was 0, a finding suggestive of monosomy X. We then verified these findings using fluorescence *in situ* hybridization (FISH) with centromere probes for the X and Y chromosomes. After counting 200 cell nuclei, we found an X/XX ratio of 4/196, practically ruling out a monosomy X cell line in uncultured CV (Figure 1).

**TABLE 1:** Short tandem repeat (STR) markers of QF-PCR showing the suspected abnormal chromosome X in chorionic villi and amniotic fluid, suggestive of its maternal origin.

Marker	Chorionic villus (ratio)*	Amniotic fluid	Maternal blood
DXS1187	155	155	155/159
DXS8377	209/216 (1.21:1)	209	203/209
DXS6809	268/276 (1.14:1)	268	264/268
DXS981	343/350 (0.86:1)	350	347/350

Notes: *The reference values for the ratio are 0.86–1.21:1. STR. The markers indicated that the X chromosome was only of maternal origin in the amniotic fluid sample, whereas in the chorionic villi sample, it was of both maternal and paternal origin.

Given the conflicting results of FISH, QF-PCR, and CMA with CV, along with the observation of nuchal translucency in the fetus, we could not exclude the possibility of an abnormal chromosome X. We, therefore, performed more fetal ultrasounds at 18 GW and discovered a nuchal cystic hygroma (2.6 × 0.7 × 1.9 cm in size), along with coronary sinus dilatation and persistent left superior vena cava. After our advice that AC would provide additional information about the fetus, the couple decided to provide AF samples at 20 GW. We then performed QF-PCR and CMA again, followed by FISH verification. The results clearly indicated complete X monosomy in uncultured AF.

After further counseling, the woman requested pregnancy termination in our hospital. We then obtained informed consent to perform multiple biopsies on the aborted fetus, and FISH was performed on placental, amniotic sac, umbilical cord, skin, and muscle samples. Postnatal chorionic villi samples from the placenta yielded the same result as those for the prenatal chorionic villi, while the amniotic sac exhibited mosaic monosomy X (16%), and the fetal skin, muscle, and umbilical cord samples showed near-complete monosomy X. The details are presented in Table 2.

**TABLE 2:** Results from FISH of the aborted fetus.

Tissue	Sex chromosome outcome	Tissue	Sex chromosome outcome
Placenta 1	X/XX = 2/98	Amniotic sac 1	X/XX = 29/71
Placenta 2	X/XX = 0/100	Amniotic sac 2	X/XX = 2/98
Placenta 3	X/XX = 1/99	Umbilical cord	X/XX = 98/2
Placenta 4	X/XX = 3/97	Muscle–left forearm 1	X/XX = 88/12
Placenta 5	X/XX = 6/94	Muscle–left forearm 2	X/XX = 98/2
Placenta 6	X/XX = 2/98	Skin 1	X/XX = 98/2
Placenta 7	X/XX = 2/98	Skin 2	X/XX = 97/3
Placenta 8	X/XX = 1/99		

Notes: The numbers represent different sampling locations of a given tissue in the aborted fetus.

## Discussion

Chromosomal mosaicism is an important cause of cytogenetic variation across fetal tissues. This phenomenon is a major challenge for all cytogenetic laboratories concerned with prenatal diagnosis and clinical counseling (Capalbo and Laura, 2017; Cherry et al., 2017; Lund et al., 2020; Westenius et al., 2021). Samples for genetic testing in prenatal diagnosis are obtained from CVS, AC, and FBS. In particular, CVS is widely accepted as the diagnostic sample of choice in the first trimester (Emily et al., 2018), and CPM is usually the most common form of placental mosaicism. The present case exhibited a special mosaicism and was distinct from CPM, where the AF samples revealed completely abnormal chromosome X, but the CV samples suggested only low-level mosaicism.

**FIGURE 2:**
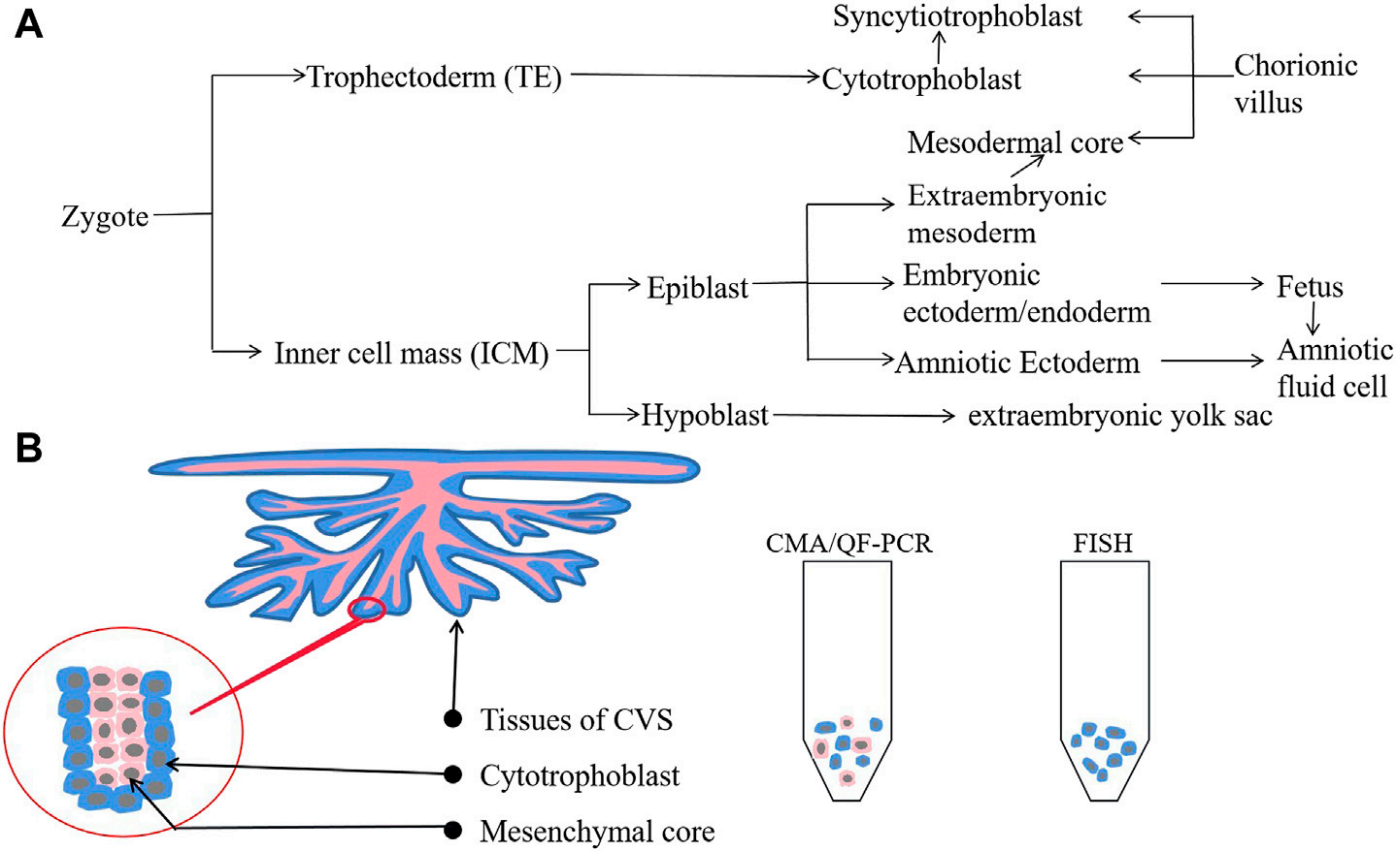
Cell lineage from zygote to fetus and populations of CVS. **(A)** Cell lineage from zygote to fetus. **(B)** Cell populations from uncultured CVS differ between CMA/QF-PCR (both cytotrophoblasts and mesenchymal core) and FISH (mainly cytotrophoblasts).

Therefore, we analyzed the cell line of CV samples in the present case. The CV is composed of three cell types: syncytiotrophoblast, cytotrophoblast, and mesodermal core (Figure 2A). Syncytiotrophoblasts develop from cytotrophoblasts of the trophectoderm, and the mesodermal core develops from the extraembryonic mesoderm of the inner cell mass. None of these lines originate directly from the fetus proper. Therefore, the distribution of mosaicism between the fetus and placental cells depends on when and where the mutation occurred (Figure 2A) (Crane and Cheung, 1988; Bianch et al., 1993; Van Den Berg et al., 2006; Boss et al., 2018). Analysis of CV mosaics has shown that the mesenchyme core contributes nearly 50% to a DNA pool derived from uncultured dissociated CVS (Mann et al., 2007). In the present case, we performed QF-PCR and CMA on genomic DNA extracted from uncultured CVS, containing a mixture of cytotrophoblast and mesenchymal-core populations (Figure 2B). The chorionic villi samples used for FISH were uncultured and treated with a dissociation solution of methanol-glacial acetic acid without digestion; thus, the cell populations used for this test were probably mainly derived from the cytotrophoblast (Figure 2B). Thus, we hypothesized that the number of X chromosomes in the syncytiotrophoblast and cytotrophoblast was normal but that mosaic or complete monosomy of chromosome X was present in the mesenchymal core.

In addition, we performed FISH on samples taken from multiple biopsy sites (placenta, umbilical cord, amniotic sac, skin, and muscle) to better understand the cause of this special case. The results confirmed our speculation that the mosaicism increased gradually from the placenta to the fetal membrane, and finally to the umbilical cord. All eight placental samples revealed near-normal chromosome X, while results from fetal skin, muscle, and umbilical cord samples were consistent with the AF results showing complete monosomy X. The amniotic sac revealed mosaicism of monosomy X. The QF-PCR and SNP results showed that the two X chromosomes were respectively of maternal and paternal origin, meaning the phenomenon could not be the result of a monomic rescue event after an error during meiosis. Instead, mitotic errors may have been the cause of the mosaic cell lines in our case, with the genetic error occurring during the early stage of the inner cell mass, similar to the conclusions of previous studies on cell differentiation in human embryos (Guichet et al., 1995; Beverstock et al., 1998; Chen et al., 2013; Westenius et al., 2021; Taylor et al., 2014).

Regarding clinical significance, our findings indicate that if CVS reveals suspicious abnormalities, further genetic testing should be performed on AF as it contains more blastoderm-derived fetal cells and, thus, provides more information. Traditional karyotype analysis following cell culture targets cells from the mesenchymal core, which has been shown to be a very reliable predictor of fetal karyotype, whereas more recent molecular techniques are thought to test both mesenchymal core and cytotrophoblast (Mann et al., 2007). Moreover, the advent of newer molecular cytogenomic technologies such as CMA has brought about the prospect of greater diagnostic resolution than conventional cytogenetic methods. For over a decade, CMA has been broadly offered when multiple fetal malformations are detected including NT ≥ 3.5 mm (Wapner et al., 2012; Armour et al., 2018; Riggs et al., 2020). However, CMA and QF-PCR may not be sensitive enough to detect low-level mosaicism for aneuploidy, which can detect mosaicism as low as approximately 10%–20%, while FISH provides greater accuracy when estimating chromosome mosaicism (detection limit as low as ≤10%) as it can count more cells. Therefore, FISH is the more suitable method for unusual cases like the present case. Moreover, it is also important to consider the cell lines we test as this is also an important factor in avoiding false-negative or false-positive findings in clinical genetics (Figure 2B). Therefore, we recommend that multiple tests be performed simultaneously to reduce the risk of misdiagnosis in prenatal diagnosis.

Nevertheless, all our selected samples and methods have limitations, which should be considered to minimize false-negative or false-positive findings during diagnostic testing. Another important direction for future research is a comprehensive exploration of chromosome types across different fetal tissues, along with an investigation of the mechanisms underlying chromosomal abnormalities from the perspective of embryonic cell differentiation. The combination of improved detection technology and clarity on cell differentiation mechanisms should improve the accuracy of clinical genetic diagnoses and help with prenatal decision-making.

In conclusion, we recommend that prenatal diagnosis employs multiple, simultaneous techniques to reduce the risk of misdiagnosis. Prenatal consultation should combine genetic testing with ultrasounds to verify the phenotype to ensure a comprehensive evaluation of fetal health.

## Data Availability

The datasets for this article are not publicly available due to concerns regarding participant/patient anonymity. Requests to access the datasets should be directed to the corresponding authors.

## Supplementary material

SUPPLEMENTARY FIGURE S1 FISH results of the aborted fetus. The blue signal is chromosome 18, and the green signal is chromosome X.
