# Supplementary figures and images for: Case report: Prenatal diagnosis of rare chromosome mosaicism: discordant results between chorionic villi and amniotic fluid samples
Source: Front Genet. 2023 Jun 5;14:1165019. doi: 10.3389/fgene.2023.1165019 (PMC10277675; doi:10.3389/fgene.2023.1165019)

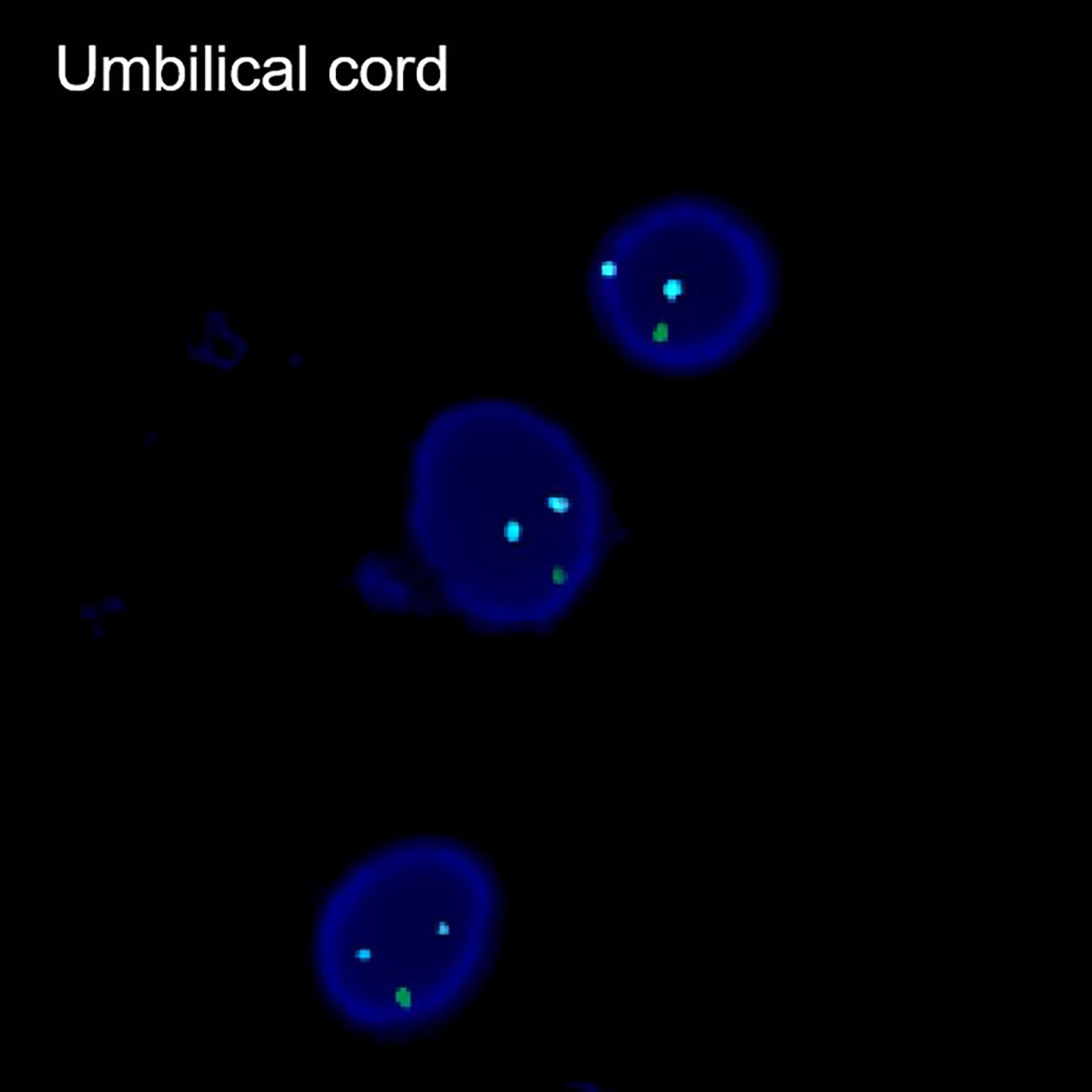

Supplement: Supplementary file 1 [file Image5.jpg]

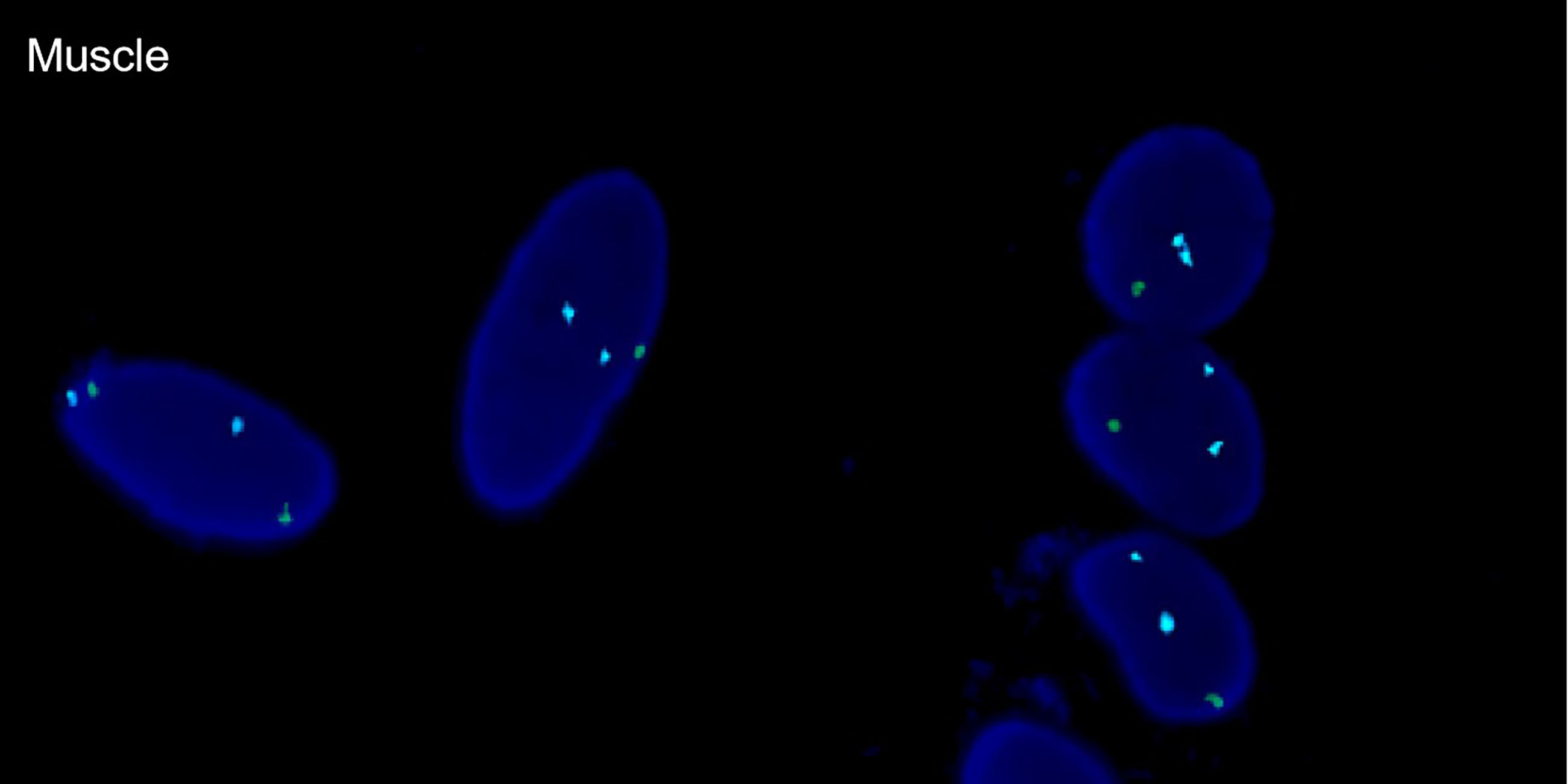

Supplement: Supplementary file 2 [file Image6.jpg]

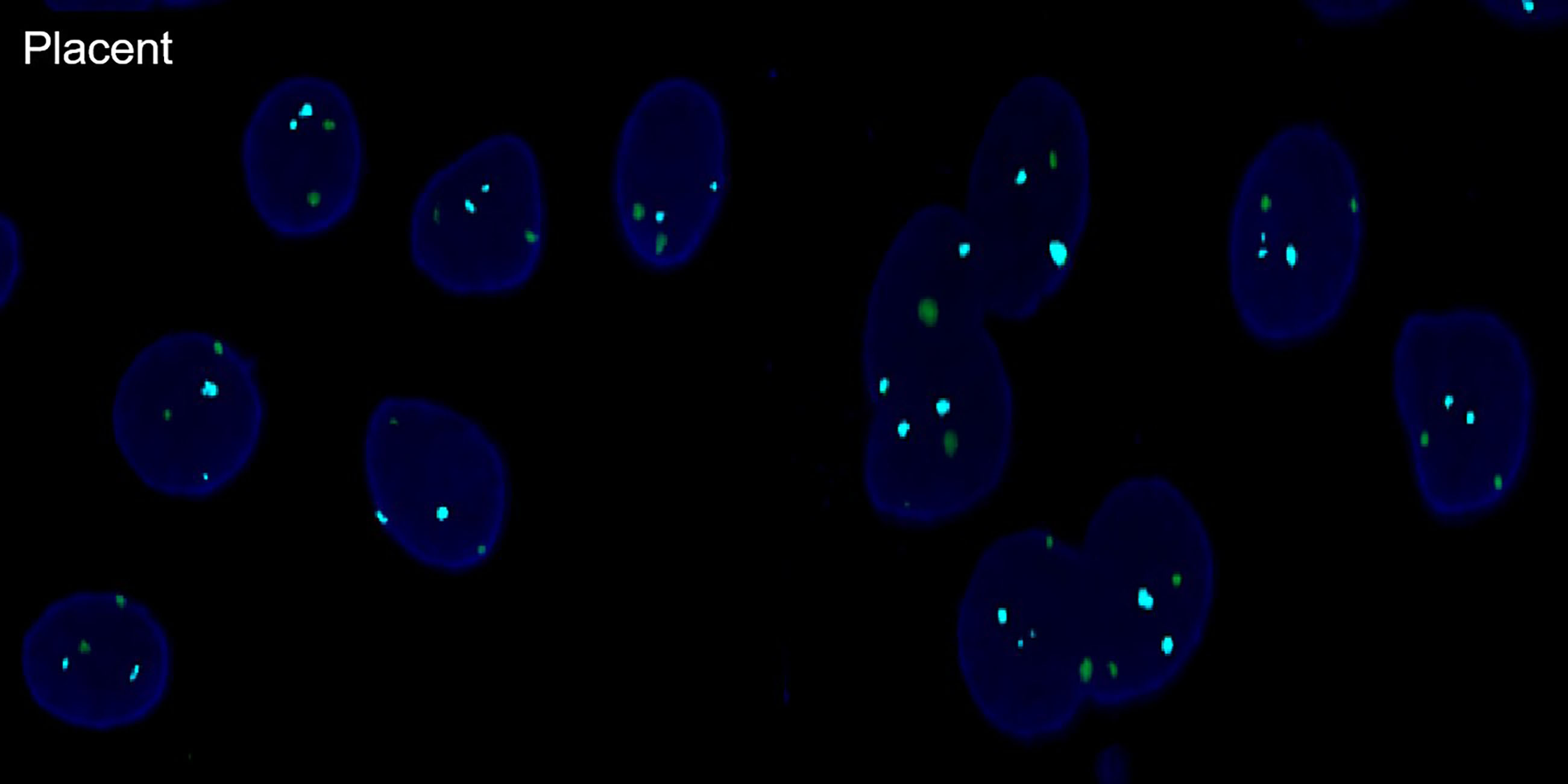

Supplement: Supplementary file 3 [file Image3.jpg]

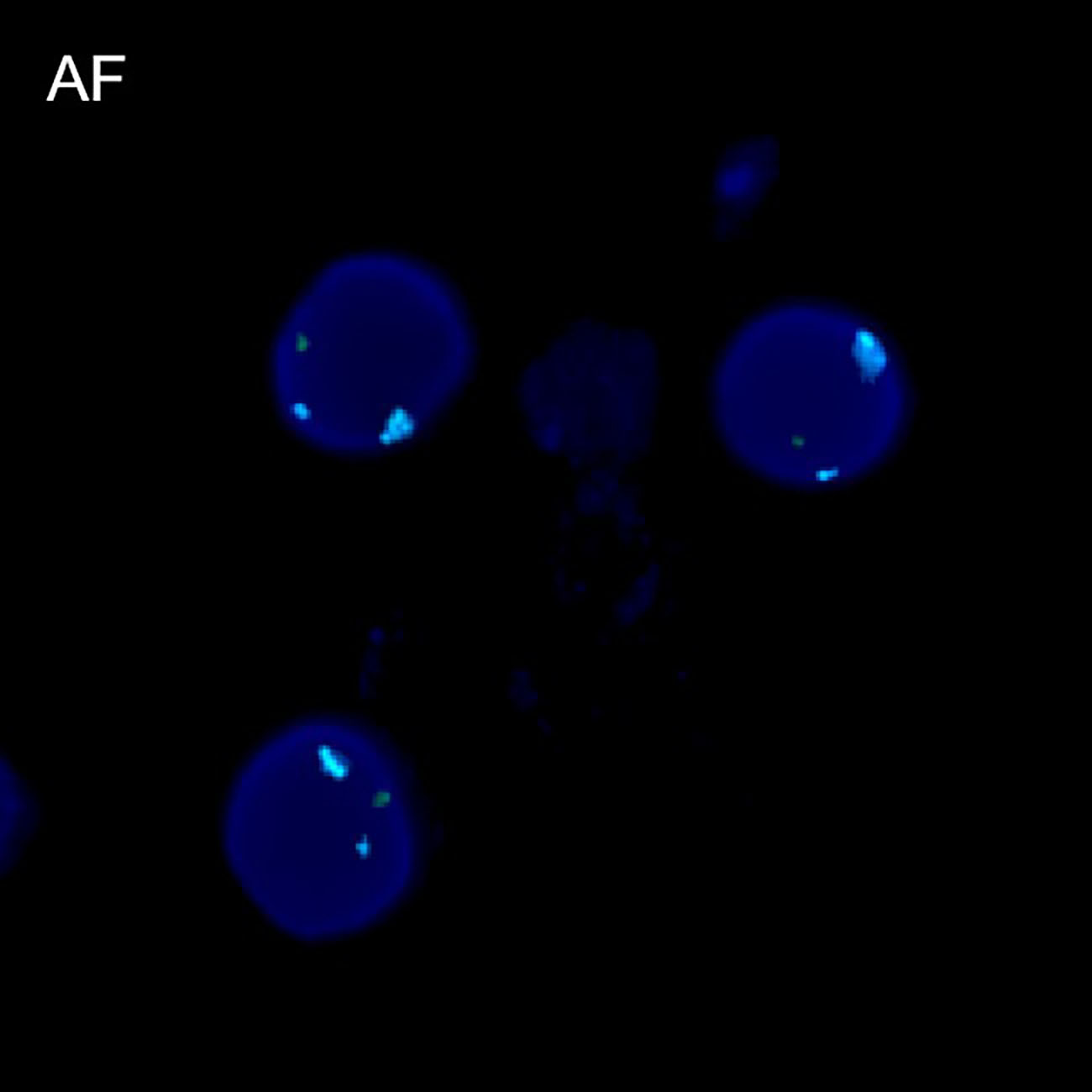

Supplement: Supplementary file 4 [file Image2.jpg]

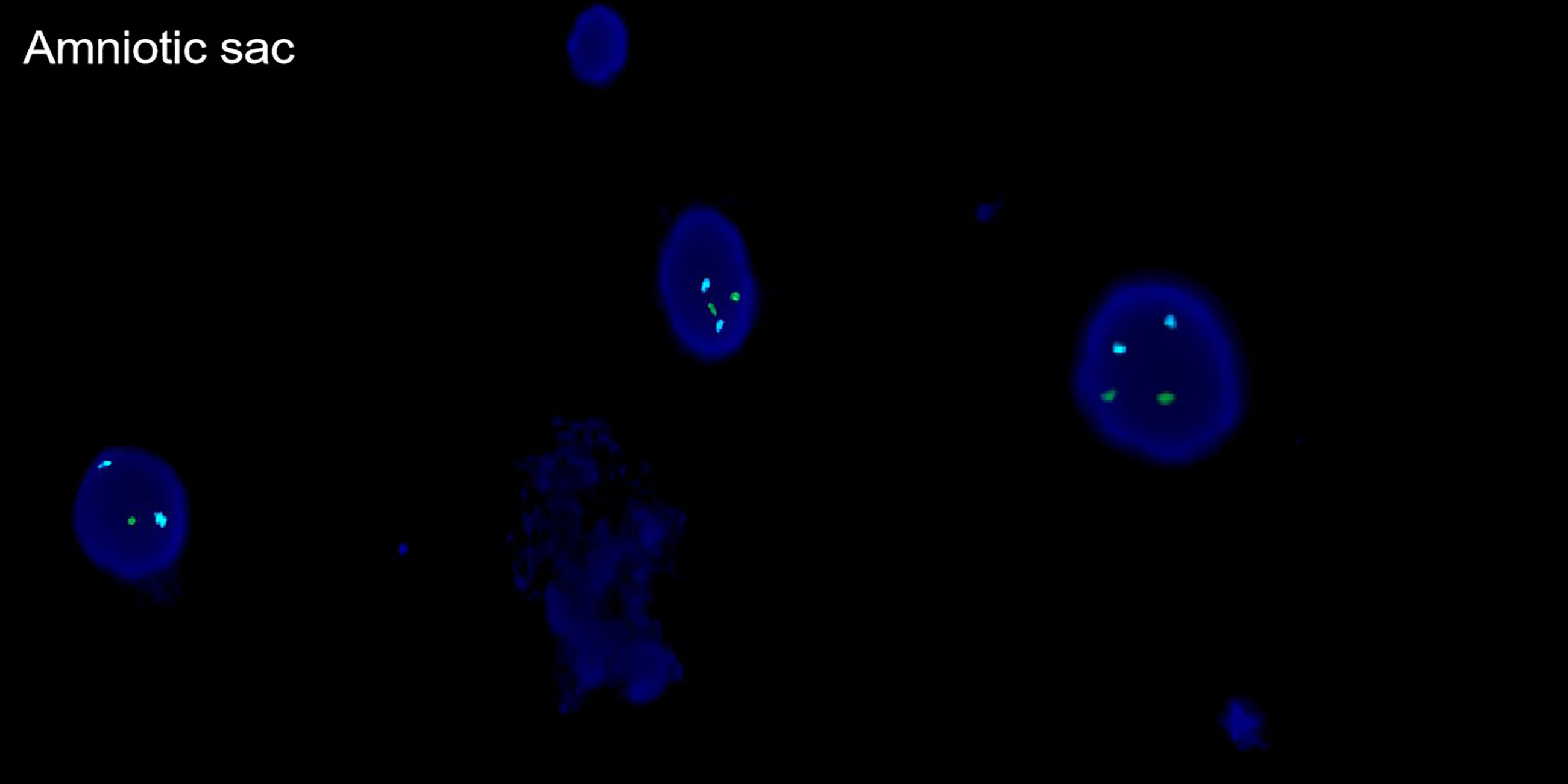

Supplement: Supplementary file 5 [file Image4.jpg]

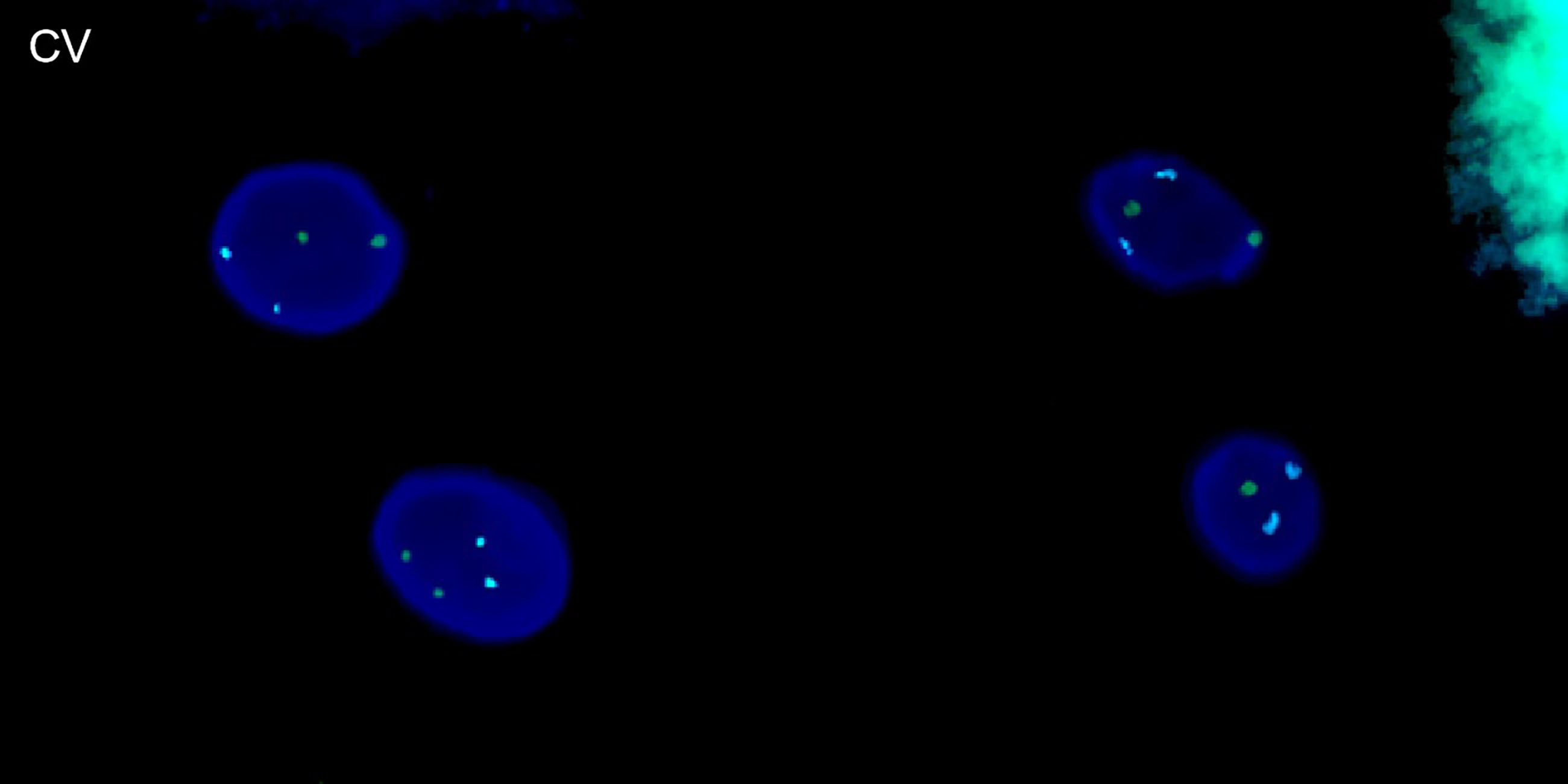

Supplement: Supplementary file 6 [file Image1.jpg]
